# Affective bias and current, past and future adolescent depression: A familial high risk study

**DOI:** 10.1016/j.jad.2014.11.046

**Published:** 2015-03-15

**Authors:** Emma J. Kilford, Lucy Foulkes, Robert Potter, Stephan Collishaw, Anita Thapar, Frances Rice

**Affiliations:** ^a^Department of Clinical, Educational and Health Psychology, University College London, 26 Bedford Way, London WC1H 0AP, United Kingdom; b Institute of Psychological Medicine and Clinical Neurosciences, MRC Centre for Neuropsychiatric Genetics and Genomics, Cardiff University, Cardiff, United Kingdom

**Keywords:** Depression, Adolescence, High-risk, Cognitive, Affective, Longitudinal

## Abstract

**Background:**

Affective bias is a common feature of depressive disorder. However, a lack of longitudinal studies means that the temporal relationship between affective bias and depression is not well understood. One group where studies of affective bias may be particularly warranted is the adolescent offspring of depressed parents, given observations of high rates of depression and a severe and impairing course of disorder in this group.

**Methods:**

A two wave panel design was used in which adolescent offspring of parents with recurrent depression completed a behavioural task assessing affective bias (The Affective Go/No Go Task) and a psychiatric interview. The affective processing of adolescents with current, prior and future depressive disorder was compared to that of adolescents free from disorder.

**Results:**

Adolescents with current depression and those who developed depression at follow-up made more commission errors for sad than happy targets compared to adolescents free from disorder. There was no effect of prior depression on later affective processing.

**Limitations:**

Small cell sizes meant we were unable to separately compare those with new onset and recurrent depressive disorder.

**Conclusions:**

Valence-specific errors in behavioural inhibition index future vulnerability to depression in adolescents already at increased risk and may represent a measure of affective control. Currently depressed adolescents show a similar pattern of affective bias or deficits in affective control.

## 1 Introduction

Adolescence is associated with a marked increase in the prevalence of depressive symptoms and disorder (Kim-Cohen et al., 2003; Lewinsohn et al., 1998; Thapar et al., 2012). Depression in young people is not benign and is associated with a range of poor outcomes including deliberate self-harm, academic failure and poor mental health in adulthood. Cognitive theories of depression propose that affective bias and negative styles of thinking play a crucial role in the development and maintenance of depression (Beck, 2008; Roiser et al., 2012). More recent models emphasise the role of ‘low level’ affective information processing biases in the development of ‘higher level’ negative schemata and depression (Roiser et al., 2012). Whilst it is clear that depressive symptoms and affective biases *co-occur*, the precise role of affective biases in the *onset* of depression and the role of prior depression on later affective processing is unclear (Jacobs et al., 2008; Roiser et al., 2012). Longitudinal studies are required in order to determine whether affective biases are state markers associated with current depression, or ‘trait’ markers of risk that precede depression onset or persist after remission.

One group where the investigation of affective processing and depression is particularly warranted is the offspring of depressed parents. Parental depression is a robust risk factor for depression in adolescence, with approximately 40% of the offspring of depressed parents developing depressive disorder themselves by early adulthood (Rice et al., 2002). Although there is heterogeneity in outcome for the children of depressed parents, when depression does develop, evidence suggests a severe and impairing course (Lieb et al., 2002). The potential importance of affective processing in explaining outcome in this high-risk group is illustrated by the efficacy of a preventive form of Cognitive Behavioural Therapy (CBT) that seeks to challenge negative thinking in selected high-risk groups (Garber et al., 2009), and reports of more negative explanatory styles (schemata) in high-risk compared to low-risk offspring when self-report measures are used (Garber and Robinson, 1997). However, existing studies of affective bias in adolescent depression are often cross-sectional making it difficult to draw conclusions about the direction of influence over time. Moreover, very few studies to date have used behavioural measures of affective processing which are thought to provide a more objective assessment of affective bias than self-report questionnaires, which rely on introspection and awareness of affective bias.

The Affective Go/No Go task (AGN; Murphy et al., 1999) is an inhibitory control paradigm that has been used to investigate affective biases in depressed adults and adolescents. The task requires participants to make a motor response (‘go’) to words of a target valence (happy or sad), while simultaneously inhibiting motor responses (‘no-go’) to words of the competing valence. It also involves affective set-shifting of attention and responses, as the target category changes across experimental blocks. Depressed adults have been shown to respond faster to sad targets than happy targets, and miss more happy than sad targets (Erickson et al., 2005; Murphy et al., 1999), suggesting the presence of affective biases in currently depressed adults.

Two cross-sectional studies have examined affective processing in adolescent depressive disorder using the AGN. Although these studies have found evidence of affective bias, they do not precisely mirror those reported in adult studies. Kyte et al. (2005) compared the performance of healthy controls to that of adolescents with a first onset of depression in the past year. Recently depressed adolescents made more commission errors during blocks with happy targets, suggesting they were less able to inhibit responses to sad distractors. Maalouf et al. (2012) included current and remitted depression groups as well as healthy adolescent controls. They found evidence of state-dependent affective biases; currently depressed adolescents responded more quickly when shifting to sad targets than when shifting to happy targets compared to remitted and control adolescents. To date, there is no longitudinal study of affective bias measured with the AGN and adolescent depression, and no such study in adolescents at high familial risk of developing depression.

In this study we examined affective bias in a 1-year longitudinal study of adolescents at risk of depression due to parental history of depression. The aim was to assess relationships between adolescent depressive disorder and affective bias by making use of a two-wave panel design where psychopathology and affective bias had been assessed on two occasions using well-validated methods. We examined the following questions: What is the cross-sectional and longitudinal relationship between measures of affective bias and depression in a high-risk sample? Specifically, we examined 1) the association of affective bias with current depression and 2) the relationship between earlier depression and later affective bias, in order to assess whether experience of depression alters affective processing. Finally, we examined 3) whether individuals with depression at follow-up (new onset or recurrence) differed in their affective processing at baseline from those who did not.

## 2 Method

### 2.1 Participants

Participants came from a three-wave longitudinal study of the offspring of parents with recurrent unipolar depression: the Early Prediction of Adolescent Depression (EPAD) study (Mars et al., 2012). Parents were recruited predominantly from primary care (general practice surgeries) in South Wales, UK on the basis of treatment for at least two episodes of DSM-IV major depressive disorder (confirmed at interview). The mother was the affected parent in 93% of the eligible sample at baseline and 99% reported their ethnicity as British (Mars et al., 2012). This paper reports on data collected at the second (hereafter referred to as baseline) and third assessments (carried out on average 12.5 months later; hereafter referred to as follow-up) of this cohort, when adolescents completed a test battery including the AGN. Assessments were conducted in families׳ homes. Parents and adolescents aged 16 years and over provided written informed consent, younger participants provided written assent. Ethical review and approval were provided by the Multi-Centre Research Ethics Committee for Wales.

**Fig. 1:**
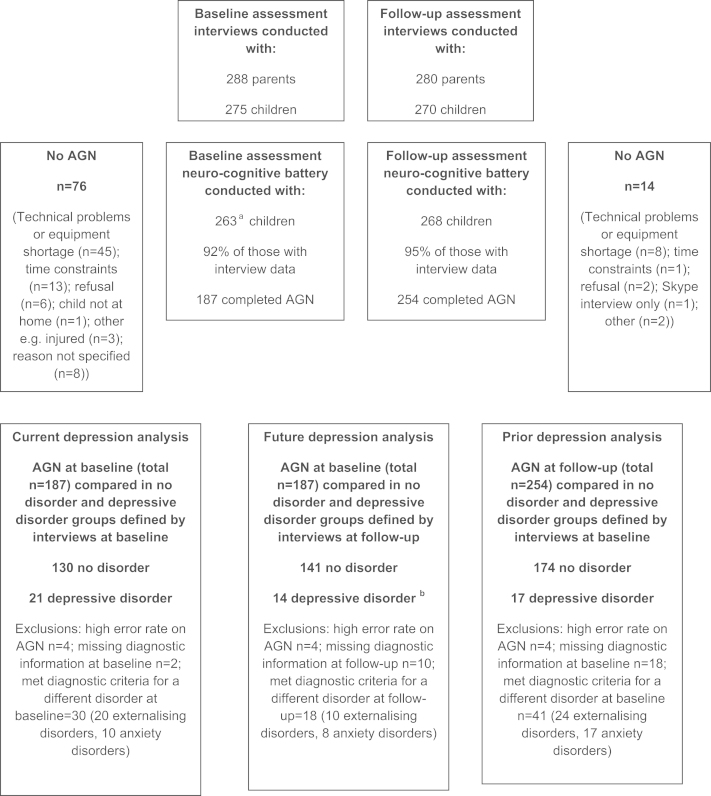
Participation details. ^**a**^Assessments were completed on 265 children but this included 2 children who were later excluded due to parental bipolar disorder. ^**b**^5 cases were new onset episodes of depressive disorder, 6 were recurrences from the baseline assessment and 3 individuals had different disorders at baseline (one individual had diagnoses of generalised anxiety disorder and disruptive behaviour disorder NOS at baseline, one had a diagnosis of obsessive compulsive disorder and one had a diagnosis of oppositional defiant disorder). Externalising disorders included diagnoses of oppositional defiant disorder, conduct disorder, disruptive disorder or ADHD (but no diagnosis of depression). Anxiety disorders included diagnoses of generalised anxiety disorder, separation anxiety, social phobia, panic disorder, agoraphobia, or obsessive–compulsive disorder (but no diagnosis of depression). Adolescents were assigned to the ‘no disorder’ group if they were free from psychopathology.

We included participants with no disorder or with depressive disorder (see Section 2.2). Fig. 1 describes participation rates, reasons for non-completion of assessments and the groups that were compared. Technical issues at baseline meant that the AGN completion rate was lower than at follow-up. Nevertheless, there was no evidence of systematic differences in participation between study phases: there were no differences between adolescents who completed the AGN and those who did not in terms of gender (*χ* ^2^ =.10, *p*=.753 baseline; *χ* ^2^ =.09, *p*=.350 follow-up) or depressive symptoms (*t*=−.07, df=282, *p*=.948 baseline; *t*(31.06)=.73, *p*=.474 follow-up), although participants completing the AGN had higher IQ scores (*t*(328)=−2.83, *p*=.005 baseline; *t*(328)=−4.32, *p*<.001 follow-up).

### 2.2 Assessments

#### 2.2.1 Emotional processing task

Participants completed the Affective Go/No Go task (AGN) task (www.camcog.com; Murphy et al., 1999) which takes approximately 10 min to administer. Sad and happy words are rapidly presented one at a time in the centre of a screen and participants are required to respond to words matching a target valence by pressing a button, while ignoring words of the other valence (distractor stimuli). The task consists of 10 blocks (2 practice and 8 experimental) of 18 words (nine happy and nine sad), each of which is presented for 300 ms, with an inter-stimulus interval of 900 ms. 45 happy words (e.g. joyful, confident) and 45 sad words (e.g. mistake, gloomy) matched for word length and frequency are presented randomly. In each block either happy (H) or sad (S) words are specified as the target valence, in one of the following randomly assigned presentation orders: HHSSHHSSHH, SSHHSSHHSS. The first two blocks are practice blocks. Of the eight experimental blocks, in four the target valence stays the same between blocks (non-shift condition), and in four the target valence changes between blocks (shift condition). In shift blocks participants are required to inhibit their previous response and respond to a new target valence, enabling assessment of set shifting and cognitive/inhibitory control. The task gives three outcome measures of interest: 1) mean reaction time to respond to target words in trials where the correct response is given (latency); 2) total number of button presses to distractor stimuli (commissions) and 3) the total number of missed responses to targets (omissions). A 500 ms/450 Hz tone sounded for commissions; however no feedback was given for omissions.

#### 2.2.2 Psychopathology and derivation of groups

Adolescent psychiatric disorders and symptoms were assessed using the Child and Adolescent Psychiatric Assessment (CAPA; Angold and Costello, 2000), which is a semi-structured interview that provides a detailed assessment of psychopathology over the previous 3 months. Interviews were conducted separately with the parent and adolescent, and a disorder was considered present if a diagnosis was made based on either interview. All cases meeting DSM-IV criteria and sub-threshold cases were reviewed by two child psychiatrists and diagnoses agreed by clinical consensus. Group comparisons in the present analyses focused on those with depressive disorder and those free from psychopathology. Participants were classified as having depressive disorder if they received a diagnosis of major depressive disorder, dysthymia, depression not otherwise specified or minor depression (2 weeks of low mood plus 1 symptom with associated incapacity). Minor depression was included in the depressed group on the basis that symptoms below the diagnostic threshold are impairing and associated with future depressive episodes (Angold et al., 1999). Participants were classified as having no disorder if they were free from psychopathology. Symptom counts of depression (possible range 0–9) and generalised anxiety (possible range 0–14) from the CAPA were also calculated. Full scale IQ was assessed using 10 subscales of the Wechsler Intelligence Scale for Children—Fourth Edition (WISC-IV; Wechsler, 2004).

In order to address the primary research question, three groups of depressed participants were formed (current, prior and future) and the affective processing of these groups was compared to that of participants with no disorder. The *current depression* analysis compared affective processing at baseline in individuals depressed or free from disorder at baseline. The *prior depression* analysis compared affective processing at follow-up in individuals depressed or free from disorder at baseline. Finally, the *future depression* analysis compared affective processing at baseline in those depressed or free from psychopathology at follow-up. Fig. 1 outlines the numbers of participants with AGN data for each of these three comparisons. Basic demographics of the groups are illustrated in Table 1; as expected, the depressed groups tended to be older and include a greater proportion of females than the no disorder groups but there were no differences between the three depression groups. AGN data were excluded where the number of missed responses was high (omissions >70%; Fig. 1). The final samples were: *Current depression* (21 depressed, 130 no disorder); *prior depression* (17 depressed, 174 no disorder); *future depression* (14 depressed, 141 no disorder). Of the 14 *future depression* cases, 5 were new onset depressive disorders, 6 were persistently depressed at baseline and follow-up and 3 cases had different disorders at baseline (Fig. 1; footnote). Group sample sizes were discrepant as would be expected in a naturalistic cohort study of this kind. However, there were no substantial group variance differences (Tabachnick and Fidell, 2001). Demographic characteristics of the analysed sub-sample at baseline were as follows: the median occupational classification of the main earner was associate professional/technical and median family income before tax was £30,000–£40,000. 60% of adolescents were living in 2-parent families, 10% in families with a parent and a step-parent and 30% in single parent families.

## 3 Results

### 3.1 Data analysis

Total commissions, omissions, and depressive and anxiety symptom counts were square root transformed prior to analysis to approximate normality; however presented means are untransformed. To assess the effect of current, prior and future depression on affective bias, mixed repeated ANOVAs were performed on each of the three AGN measures (mean correct latency, total commissions and total omissions). Diagnostic group (no disorder vs. depressive disorder) was a between-subjects factor. Within subjects factors were valence (happy vs. sad targets) and shift condition (shift vs. non-shift blocks). All analyses included gender, IQ, age and generalised anxiety symptom counts from the CAPA as covariates. Anxiety was included in order to control for potential influences of co-occurring anxiety on affective bias (e.g. Ladouceur et al., 2006). Depressive symptom counts from the CAPA were included as a covariate in the ‘future depression’ analysis in order to rule out the possibility that any observed effects stemmed from continuity of depression over time. Three-way interactions were followed up by conducting separate repeated measures ANOVAs for each diagnostic group. Two-way interactions were followed up using simple effects analysis (Howell, 1997). AGN data collected at the *baseline assessment* was used to assess the effects of *current and future depression* on affective processing. AGN data collected at the *follow-up assessment* was used to assess the effects of *prior depression* on affective bias (Fig. 1).

### 3.2 Current depression and affective bias

Table 2 illustrates results for AGN outcome measures. For latency, the depressed group responded more quickly than the no disorder group (main effect of group: *F*(1,136)=4.439, *p*=.037, *η*^2^=.032). There were no interaction effects.

For commission errors, there was a 3-way interaction among diagnostic group, valence and shift [*F* (1, 136)=5.457, *p*=.021, *η*^2^=.039]. Analysis of each group separately revealed an interaction between valence and shift in no disorder participants [*F* (1, 117)=4.46, *p*=.037, *η*^2^=.037] but not depressive disorder participants [*F*=.011]. Simple effects analysis showed that for the no disorder group, there was an influence of shift only when target valence was happy [Happy: *F* (1, 117)=4.81, *p*=.030, *η*^2^=.0391; Sad: *F*(1,117)=.248, *p*=.620, *η*^2^=.002], with more commissions made on shift trials. In contrast, set-shifting affected commission rates in depressive disorder participants only when target valence was sad [Sad: *F* (1, 15)=4.05, *p*=.062, *η*^2^=.213; Happy: *F*(1, 15)=.105, *p*=.751, *η*^2^=.007], with more commissions made on shift trials (Table 2).

There were no significant main or interaction effects of group on omission errors (Table 2).

### 3.3 Previous depression and affective bias

There were no significant main or interaction effects of group at baseline on AGN measures at follow-up for any of the three AGN measures (Table 2).

### 3.4 Future depression and affective bias

When examining depressive disorder at follow-up, there were main effects of group on both latency [*F* (1,141)=5.16, *p*=.025, *η*^2^=.035] and omission errors [*F* (1,141)=4.02, *p*=.047, *η*^2^=.028], with those meeting diagnostic criteria for depression at the follow-up assessment being faster and making more omission errors than the no disorder control group. There were no significant interaction effects with group on latency or omission errors (*F׳*s<2.42). However, there was an interaction between valence and group for commission errors [*F* (1,141)=6.09, *p*=.0156, *η*^2^=.041] when controlling for co-occurring anxiety and baseline depressive symptoms. Follow-up simple effects showed this was due to individuals with future depressive disorder making a greater number of commission errors for sad compared to happy stimuli [*F* (1,141)=8.85, *p*=.003, *η*^2^=.059]. There was a similar though much less pronounced effect of valence in individuals without a disorder (Table 2; [*F* (1,141)=1.68, *p*=.197, *η*2=.012]).

Adding socio-occupational status as an additional covariate to ANCOVAs did not alter results and demographic variables were not associated with affective bias. Finally, we examined the stability of affective biases over time (Supplementary Material). Affective biases showed moderate temporal stability (median correlation coefficient=.563), stability was similar for commission (median *r*=.564), omission (median *r*=.521) and latency (median *r*=.584) measures of affective bias and was similar for the depressed (median *r*=.576 baseline; median *r*=.569 follow-up) and no disorder (median *r*=.530 baseline; median *r*=.569 follow-up) groups.

## 4 Discussion

Our aim was to use a naturalistic longitudinal high-risk design to assess the temporal relationship between affective bias and adolescent depression. Results indicated a mood-congruent effect of current depressive disorder on affective processing and no influence of prior depression on later affective processing, which is consistent with a previous study comparing adolescents with remitted and current depressive disorder (Maalouf et al., 2012). In addition, when controlling for baseline depressive symptoms and co-occurring symptoms of generalised anxiety, adolescents who later developed depressive disorder showed baseline affective processing that was more negatively biased than those who were later free of psychopathology. This indicates that negative biases in affective processing may pre-date depressive symptoms, making this a potentially useful target for detection and prevention of future depressive disorder.

The present results suggest valence-specific effects on cognitive control that differ for adolescents who are currently depressed or free from disorder and also predict the development of depressive disorder over time. We observed group-dependent effects of valence on commission errors (on shift trials) whereby currently depressed individuals showed a greater number of errors for sad targets and healthy individuals showed a greater number of errors for happy targets. This perhaps indicates that sad stimuli interfere with cognitive control (i.e. result in a greater number of errors in behavioural inhibition) in depressed individuals while happy stimuli are interfering in adolescents with no disorder. Findings consistent with this interpretation are evidence of a bias for positive (happy faces) compared to negative targets (sad faces) shown by quicker reaction times and greater commission errors in healthy individuals (Schultz et al., 2007), and a pattern of neural activation consistent with greater arousal for happy compared to sad targets in healthy controls (Elliott et al., 2000). Furthermore, greater activation in dorsolateral prefrontal cortex has been reported for sad targets in depressed adults and for neutral targets in healthy controls (Elliott et al., 2002) which is consistent with the suggestion that there may be depression dependent valence-specific effects on behavioural action and inhibition. The present study indicated that the observed valence-specific effect on commission errors also appeared to index vulnerability to *later* depression. Thus, a greater number of commission errors to sad than happy targets differentiated adolescents with depressive disorder at follow-up from those free from disorder at follow-up indicating that this may be a cognitive risk marker for future depressive disorder. The stability of affective biases over time was similar for depressed and no disorder groups.

It is worth noting some differences in the present pattern of results to those reported in previous cross-sectional studies of adolescent depression. In particular, the two previous studies that have used this task to assess affective bias in adolescent depression reported results consistent with a difficulty in disengaging from sad stimuli as opposed to an interference effect of sad stimuli as reported in the present study (Kyte et al., 2005; Maalouf et al., 2012). In contrast, our results suggest that sad targets may result in an interruption in cognitive control, leading to greater commission errors for sad compared to happy targets in those who are currently depressed or become depressed at the 1-year follow-up. It is possible that differences between the samples may partly explain differences in findings. In particular, the age range in the current sample was wide and the mean age was lower than that of the two previous studies, thus it seems likely that the participants in our study found the task more difficult. This is reflected in the higher error rates seen in our sample. The present sample included only adolescents at familial risk of developing depression due to recurrent parental unipolar depression, which may limit the generalisability of findings to other samples. Indeed, this may have made this study more conservative as all participants were at increased risk of developing depression compared to the general population. Small cell sizes meant it was not possible to separately examine the influence of recurrent and new onset depressive disorders in the analysis of ‘future depression’. However, the inclusion of prior symptoms as a covariate will have partially addressed this limitation. We included minor depression in the depressed group. However, results were similar when excluding these cases (results available from last author). As we did not assess lifetime diagnoses (and instead assessed current psychopathology on two occasions), we may have missed depressive episodes in some individuals classified as unaffected. However, this would serve to make analyses more conservative. The choice to assess current rather than lifetime psychopathology is warranted given the superior reliability of the former approach (Hardt and Rutter, 2004; Moffitt et al., 2010). We also conducted nine statistical tests (three AGN measures comparing control to the three depression groups) and, as recommended by Rothman (1990, 2013), did not correct for multiple comparisons. Taken together, results are consistent with valence-specific effects on cognitive control in adolescents with current depressive disorder and those who later develop depressive disorder. This is the first demonstration that a measure of affective bias derived from a behavioural task indexes future vulnerability to adolescent depression.

## Role of funding source

This research was funded by grants from the Medical Research Council and British Academy (PI Frances Rice). The EPAD study was supported by The Sir Jules Thorn Charitable Trust (PI Anita Thapar). SC is supported by the Waterloo Foundation. EJK and LF are supported by MRC studentships. The sponsors had no role in the study design, data collection, analysis or interpretation of data.

## Conflict of interest

The authors have no conflicts of interest.

## Figures and Tables

**Table 1:** Basic demographics of the diagnostic groups.

**Compares**	**Current depression**		**Prior depression**		**Future depression**	
	Diagnostic groups (at baseline) on their AGN performance at baseline		Diagnostic groups (at baseline) on their AGN performance at follow-up		Diagnostic groups (at follow-up) on their AGN performance at baseline	
	**No disorder**	**Depressed**	**No disorder**	**Depressed**	**No disorder**	**Depressed**
**Number of participants**	130	21	174	17	141	14^a^
**% Female**	58	81	59	88	59	71
**Age at baseline**						
**Mean**	13.52	14.52	13.54	14.65	13.64	14.79
**SD**	2.02	2.27	1.96	2.12	2.03	1.63
**Range**	(10, 18)	(10, 17)	(10, 18)	(11, 17)	(10, 18)	(12, 17)
**Depressive symptom count**						
**Mean**	1.09	5.24	1.13	4.82	1.25	5.21
**SD**	1.05	1.92	1.04	1.67	1.24	2.15
**Generalised anxiety symptom count**						
**Mean**	1.02	5.05	1.07	5.29	1.05	4.69
**SD**	1.39	2.96	1.49	3.04	1.48	2.63

Symptom counts pertain to the assessment phase when participants in the depression groups met DSM-IV diagnostic criteria for depressive disorder (i.e. baseline for current and prior groups; follow-up for future group). A larger number of participants completed the AGN at follow-up hence the larger number of participants included in the prior depression analysis (which compared AGN data at follow-up in those without disorder and with depressive disorder at baseline)

a 5 cases were new onset episodes of depressive disorder, 6 were recurrences from the baseline assessment and 3 individuals had different disorders at baseline.

**Table 2:** Affective bias and associations with current, prior and future depression.

	**Current depression**		**Prior depression**		**Future depression**	
	No disorder Mean (SD)	Depressed Mean (SD)	No disorder Mean (SD)	Depressed Mean (SD)	No disorder Mean (SD)	Depressed Mean (SD)
**Latency**						
Happy target	491.93	473.92	493.38	504.18	494.47	458.63
	(95.33)	(86.06)	(90.41)	(67.74)	(93.73)	(88.90)
Sad target	499.03	463.64	499.94	512.43	500.18	447.44
	(95.45)	(97.90)	(94.46)	(74.66)	(93.32)	(104.33)
Shift to happy	485.33	462.50	488.36	493.20	488.95	444.10
	(100.85)	(92.46)	(92.97)	(65.86)	(99.53)	(90.75)
Shift to sad	493.07	474.25	497.68	509.34	494.30	440.63
	(97.71)	(112.33)	(97.91)	(80.20)	(96.53)	(110.82)
Non-shift happy	496.61	484.34	497.72	515.86	498.74	471.89
	(98.25)	(84.89)	(95.16)	(78.62)	(97.40)	(98.53)
Non-shift sad	503.07	455.25	502.11	517.20	504.72	449.21
	(103.92)	(101.51)	(98.81)	(75.29)	(102.97)	(113.46)
Group	*F*=4.439⁎		*F*=.008		*F*=5.162⁎*	
Group×valence	*F*=.946		*F*=.806		*F*=2.422	
Group×shift	*F*=.004		*F*=.792		*F*=.678	
Group×valence×shift	*F*=2.560		*F*=.392		*F*=.024	

**Commissions**						
Happy target	9.12	9.45	7.66	5.81	9.30	7.92
	(6.44)	(6.44)	(5.82)	(4.50)	(6.34)	(5.95)
Sad target	9.98	10.55	8.26	6.65	10.01	10.15
	(6.63)	(6.89)	(6.54)	(4.72)	(6.70)	(6.15)
Shift to happy	4.85	4.65	4.09	3.35	4.95	3.77
	(3.48)	(3.38)	(3.24)	(2.64)	(3.35)	(2.83)
Shift to sad	5.00	5.95	4.31	3.65	5.12	4.92
	(3.45)	(3.87)	(3.57)	(2.83)	(3.46)	(3.23)
Non-shift happy	4.27	4.80	3.57	2.47	4.36	4.15
	(3.40)	(3.41)	(2.97)	(2.24)	(3.40)	(3.44)
Non-shift sad	4.98	4.60	3.94	3.00	4.90	5.23
	(3.65)	(3.36)	(3.37)	(2.21)	(3.69)	(3.42)
Group	*F*=.732		*F*=.258		*F*=.056	
Group×valence	*F*=.067		*F*=.042		*F*=6.088⁎	
Group×shift	*F*=.022		*F*=.014		*F*=2.874	
Group×valence×shift	*F*=5.457⁎		*F*=1.697		*F*=.000	

**Omissions**						
Happy target	8.16	7.90	6.49	6.35	8.01	11.08
	(6.19)	(5.29)	(5.34)	(5.72)	(6.24)	(6.37)
Sad target	7.19	9.15	6.21	4.82	7.13	10.15
	(6.14)	(6.85)	(5.94)	(4.05)	(6.06)	(6.47)
Shift to happy	4.17	3.90	3.22	3.00	4.06	5.85
	(3.40)	(3.14)	(2.99)	(2.78)	(3.53)	(3.65)
Shift to sad	3.53	4.60	3.13	2.35	3.58	5.15
	(3.24)	(4.10)	(3.23)	(2.62)	(3.27)	(3.89)
Non-shift happy	4.20	3.40	3.26	2.47	4.07	5.85
	(3.50)	(2.39)	(2.82)	(2.48)	(3.42)	(3.16)
Non-shift sad	3.66	4.55	3.08	2.47	3.55	5.00
	(3.32)	(3.53)	(3.12)	(2.35)	(3.27)	(3.44)
Group	*F*=.209		*F*=2.637		*F*=4.019⁎	
Group×valence	*F*=1.854		*F*=.007		*F*=.762	
Group×shift	*F*=.330		*F*=.319		*F*=.382	
Group×valence×shift	*F*=1.042		*F*=.623		*F*=.290	

The total sample size varies slightly due to missing scores on covariates (IQ or symptom scores) for some participants.

⁎ *p*<.05.
