# Supplementary material for: Affective bias and current, past and future adolescent depression: A familial high risk study
Source: J Affect Disord. 2015 Mar 15;174:265–71. doi: 10.1016/j.jad.2014.11.046 (PMC4351191; doi:10.1016/j.jad.2014.11.046)
Supplement: Supplementary file 1 — Supplementary data [file mmc1.docx]

**Supplementary Material: Stability of affective processing from baseline to follow up separately for depressed and no disorder groups**

|  | **Commissions (shift negative)** | **Commissions (non-shift negative)** | **Commissions (shift positive)** | **Commissions (non-shift positive)** | **Omissions (shift negative)** | **Omissions (non-shift negative)** | **Omissions (shift positive)** | **Omissions (non-shift positive)** | **Latency (shift negative)** | **Latency (non-shift negative)** | **Latency (shift positive)** | **Latency (non-shift positive)** |
| --- | --- | --- | --- | --- | --- | --- | --- | --- | --- | --- | --- | --- |
| **Stability Depressed at wave 2 (n=15)** | **.605 *** | **.546 *** | **.612 *** | **.525 *** | **.307** | **.386** | **.606 *** | **.323** | **.454 ^a^** | **.509 ^a^** | **.519 *** | **.690 **** |
| **Stability no disorder at wave 2 (n=118)** | **.540 ***** | **.549 ***** | **.525 ***** | **.460 ***** | **.528 ***** | **.565 ***** | **.552 ***** | **.521 ***** | **.597 ***** | **.588 ***** | **.625 ***** | **.705 ***** |
| **Stability Depressed at wave 3 (n=12)** | **.574 a** | **.588 *** | **.567 a** | **.825 ***** | **.629 *** | **.709 **** | **.231** | **.747 **** | **.386** | **.629 *** | **.467** | **.572 ^a^** |
| **Stability no disorder wave 3 (n=129)** | **.561 ***** | **.586 ***** | **.576 ***** | **.440 ***** | **.520 ***** | **.512 ***** | **.516 ***** | **.490 ***** | **.568 ***** | **.580 ***** | **.604 ***** | **.701 ***** |

^a^ p<.1, * p<.05; ** p<.01; *** p<.001
